# Correlation of Minimum Apparent Diffusion Coefficient and Maximum Standardized Uptake Value of the Primary Tumor with Clinicopathologic Characteristics in Endometrial Cancer

**DOI:** 10.4274/mirt.30306

**Published:** 2017-02-01

**Authors:** Evrim Sürer Budak, Tayfun Toptaş, Funda Aydın, Ali Ozan Öner, Can Çevikol, Tayup Şimşek

**Affiliations:** 1 Antalya Training and Research Hospital, Clinic of Nuclear Medicine, Antalya, Turkey; 2 Akdeniz University Faculty of Medicine, Department of Gynecologic Oncological Surgery, Antalya, Turkey; 3 Akdeniz University Faculty of Medicine, Department of Nuclear Medicine, Antalya, Turkey; 4 Afyon Kocatepe University Faculty of Medicine, Department of Nuclear Medicine, Afyonkarahisar, Turkey; 5 Akdeniz University Faculty of Medicine, Department of Radiology, Antalya, Turkey

**Keywords:** Endometrial cancer, maximum standardized uptake value, minimum apparent diffusion coefficient

## Abstract

**Objective:**

To explore the correlation of the primary tumor’s maximum standardized uptake value (SUV_max_) and minimum apparent diffusion coefficient (ADC_min_) with clinicopathologic features, and to determine their predictive power in endometrial cancer (EC).

**Methods:**

A total of 45 patients who had undergone staging surgery after a preoperative evaluation with ^18^F-fluorodeoxyglucose (FDG) positron emission tomography/computerized tomography (PET/CT) and diffusion-weighted magnetic resonance imaging (DW-MRI) were included in a prospective case-series study with planned data collection. Multiple linear regression analysis was used to determine the correlations between the study variables.

**Results:**

The mean ADC_min_ and SUV_max_ values were determined as 0.72±0.22 and 16.54±8.73, respectively. A univariate analysis identified age, myometrial invasion (MI) and lymphovascular space involvement (LVSI) as the potential factors associated with ADC_min_ while it identified age, stage, tumor size, MI, LVSI and number of metastatic lymph nodes as the potential variables correlated to SUV_max_. In multivariate analysis, on the other hand, MI was the only significant variable that correlated with ADC_min_ (p=0.007) and SUV_max_ (p=0.024). Deep MI was best predicted by an ADC_min_ cutoff value of ≤0.77 [93.7% sensitivity, 48.2% specificity, and 93.0% negative predictive value (NPV)] and SUV_max_ cutoff value of >20.5 (62.5% sensitivity, 86.2% specificity, and 81.0% NPV); however, the two diagnostic tests were not significantly different (p=0.266).

**Conclusion:**

Among clinicopathologic features, only MI was independently correlated with SUV_max_ and ADC_min_. However, the routine use of ^18^F-FDG PET/CT or DW-MRI cannot be recommended at the moment due to less than ideal predictive performances of both parameters.

## Abstract (tr)

**Amaç:**

Endometrium kanserinde (EK) primer tümörün maksimum standardize tutulum değeri (SUV_maks_) ve minimum görünen difüzyon katsayısının (ADC_min_) klinikopatolojik özellikler ile olan ilişkisini araştırmak ve bunların öngörü gücünü belirlemektir.

**Yöntem:**

^18^F-fluorodeoksiglukoz (FDG) pozitron emisyon tomografisi/bilgisayarlı tomografi (PET/BT) ve difüzyon ağırlıklı-manyetik rezonans görüntüleme (DA-MRG) ile preoperatif değerlendirmeyi takiben evreleme cerrahisi yapılan toplamda 45 hasta, planlı veri toplama yapılan prospektif bir olgu serisine dahil edildiler. Çalışma verileri arasındaki ilişkiler çoklu doğrusal regresyon analizi ile araştırıldı.

**Bulgular:**

Ortalama ADC_min_ ve SUV_maks_ sırasıyla 0,72±0,22 ve 16,54±8,73 olarak bulundu. Tek değişkenli analizde yaş, myometriyal invazyon (Mİ) ve lenfovasküler alan tutulumu (LVAT) ADC_min_ ile ilişkili potansiyel faktörler olarak bulunurken, yaş, evre, tümör büyüklüğü, LVAT ve metastatik lenf düğümlerinin sayısı SUV_maks_ ile ilişkili potansiyel değişkenler olarak tespit edildiler. Diğer taraftan, çok değişkenli analizde Mİ, ADC_min_ (p=0,007) ve SUV_maks_ (p=0,024) ile ilişkili tek anlamlı değişkendi. Derin Mİ en iyi, ≤0,77’lik [%93,7 duyarlılık, %48,2 özgüllük ve %93,0 negatif öngörü değeri (NPD)] bir ADC_min_ kesim değeri ve >20,5’lik (%62,5 duyarlılık, %86,2 özgüllük ve %81,0 NPD) bir SUV_maks_ kesim değeri ile öngörülebiliyordu. Ne var ki, her iki tanısal test birbirlerinden anlamlı şekilde farklı değildi (p=0,266).

**Sonuç:**

Klinikopatolojik özelliklerden yalnızca Mİ bağımsız ve anlamlı şekilde SUV_maks_ ve ADC_min_ ile ilişkiliydi. Ne var ki, her iki parametrenin ideal olmayan öngörü performansları nedeniyle ^18^F-FDG PET/BT veya DA-MRG’nin rutin kullanımı şu noktada önerilemez.

## INTRODUCTION

Endometrial cancer (EC) is the most common gynecologic malignancy in developed countries (1). The majority of patients present with disease limited to the uterus at the time of diagnosis, which leads to a generally high survival rate (2). Unfortunately, it has been reported that deaths from EC have increased over the past two decades, probably due to underestimation of actual tumor spread and increased rate of high-risk histology (3).

EC is staged surgically using the International Federation of Gynecology and Obstetrics (FIGO) and American Joint Committee on Cancer staging systems (4,5). While total hysterectomy and bilateral salpingo-oophorectomy (TH/BSO) is the mainstay treatment of uterine-confined disease, a comprehensive staging surgery including systematic lymphadenectomy allows for assessing the true extent of disease and the need for adjuvant therapy (6). Nevertheless, a systematic lymphadenectomy leads to a doubling of the complication rate (7). Besides, there are two randomized controlled trials demonstrating no survival benefit for lymphadenectomy especially in patients with presumed uterine-confined disease (8,9).

According to the widely agreed view, a systematic lymphadenectomy may be omitted in selected patients considered to be at low-risk for extrauterine spread, without an unfavorable impact on disease prognosis. The most used criteria for defining low-risk patients are based on preoperative and intraoperative pathologic findings including well or moderately differentiated histology, tumor size less than 2 cm, and myometrial invasion (MI) less than 50% (10). However, accurate identification of this group of patients may be somewhat problematic due to the variability in tumor grade and depth of MI on final pathologic examination (11).

The role of preoperative imaging for predicting tumor characteristics in patients with EC has been established by several studies, using different modalities (12,13,14). Diffusion-weighted magnetic resonance imaging (DW-MRI) and ^18^F-fluorodeoxyglucose (FDG) positron emission tomography combined with computed tomography (PET/CT) are the two imaging techniques that stood out from the others with their capability to provide metabolic and functional information regarding tumor tissue properties, in addition to anatomic information. Minimum apparent diffusion coefficient value (ADC_min_) derived from DW-MRI and maximum standardized uptake value (SUV_max_) derived from ^18^F-FDG PET/CT are semi-quantitative imaging biomarkers which have been suggested to be of value in estimation of tumor behavior, as well as disease prognosis (13,14). However, the clinical data regarding direct comparison of both biomarkers in preoperative evaluation of EC patients are sparse, and the routine use of them remains controversial.

In the current study, we aimed to investigate relationships of SUV_max_ and ADC_min_ of the primary tumor to clinicopathologic features, and to compare their predictive ability in patients with EC.

## MATERIALS AND METHODS

This prospective case-series with planned data collection enrolled consecutive patients with EC, who underwent primary staging surgery following a preoperative evaluation with ^18^F-FDG PET/CT and DW-MRI between May 2012 and December 2014. All imaging studies were performed within two weeks before the day of surgery, and all patients provided written informed consent.

Radiologic, pathologic and clinical data including age at surgery, ADC_min_ and SUV_max_ of the primary tumor, date and extent of the surgical procedure, number of lymph nodes (LNs) removed, stage of the disease, tumor histotype, tumor grade, tumor size, depth of myometrial invasion, lymphovascular space involvement (LVSI), cervical invasion, adnexal invasion, LN involvement, number of metastatic LNs, adjuvant therapy, disease status after primary therapy, disease recurrence, survival status, and the date of the last follow-up were recorded for all patients, following the The study were approved by the Akdeniz University of Local Ethics Committee (Protocol number: 23.12.2015; 386).

Patients with uterine sarcoma, primary synchronous malignancy, insufficient data, or that received radiotherapy, chemotherapy, or hormonal therapy as primary or neoadjuvant therapy were excluded.

### Positron Emission Tomography/Computerized Tomography Technique and Image Analysis

Patients were requested to fast for at least six hours before imaging, and a venous blood glucose level below 200 mg/dL was ensured. An oral contrast agent was administered to all patients prior to scanning. In order to facilitate urinary excretion, they were asked to drink 500 ml of water and to void just before the acquisition. A whole body acquisition using integrated PET/CT scanner (Biograph 16 LSO; Siemens, Erlangen, Germany) was performed 45 to 60 minutes after intravenous administration of ^18^F-FDG (0.16 mCi/kg). A CT scan (slice thickness, 3 mm; peak voltage, 120 kV; tube current, 110 mA/s) was performed, and used for anatomical localization and calculation of attenuation correction. The PET data were acquired from the vertex to the upper thigh, and the acquisition time for PET was three minutes per bed position. Attenuation-corrected PET, CT and fusion PET/CT images were interpreted by experienced nuclear medicine specialists. The SUV_max_ of the primary tumor was measured with a region of interest (ROI) technique. The measurements were performed in correlation with CT images while limiting the area of activity precisely and minimalizing the partial volume effect. Because of the close location to the bladder, it was important to separate the primary tumor and bladder activity in order to avoid incorrect measurements. From ROIs delineated on successive sections, the greatest SUV_max_ was noted. SUV_max_ values were automatically provided by a computer-assisted software program and they were calculated using the standard formula.

### Diffusion-Weighted Magnetic Resonance Imaging Technique and Image Analysis

MRI examinations were performed using a 1.5 Tesla MRI scanner (Avanto; Siemens, Erlangen, Germany). The imaging protocol included: T2-weighted (T2W) fast-spin-echo (FSE) imaging in the sagittal and axial planes; FSE T1-weighted (T1W) imaging in the axial plane; fat-saturated FSE T2W imaging in the coronal plane; and DW imaging in the same sagittal plane (repetition time, 6100 msec; echo time, 88 msec; flip angle, 90°; field of view, 241x329 mm; slice thickness, 4 mm; interslice gap, 0.8 mm; matrix, 240x328; number of excitations, 5). The b-values of the diffusion sensitizing gradient were 50, 400 and 800 sec/mm^2^. Post-contrast fat-saturated T1W sagittal and axial images were also obtained. Assessment of the images was performed by an experienced radiologist. The presence and the size of the endometrial lesion and its signal intensity relative to that of the adjacent myometrium were evaluated on T2W and DW images with a b-value of 800 sec/mm^2^. ADC maps were generated automatically, and the measurements were performed by placing a ROI over the endometrial lesion with paying attention not to include areas of necrosis.

### Surgical Procedures, Adjuvant Therapy and Follow-up

All patients underwent a staging surgery including at least TH/BSO, pelvic lymphadenectomy, omental biopsy, and peritoneal cytology. The pelvic lymphadenectomy consisted of complete removal of the LNs from the internal iliac, external iliac, obturatory and common iliac regions. A paraaortic LN dissection up to the renal vessels was added to the staging procedure in the presence of any of the followings:

1) Non-endometrioid or grade 2-3 endometrioid histology on preoperative biopsy,

2) MI greater than 50% on intraoperative frozen-section examination. All procedures were performed by two experienced gynecologic oncologists.

Tumor grading was conducted according to that of the World Health Organization (15), and staging was classified using the FIGO_2009_ system (4). Non-endometrioid histotypes were considered grade 3 tumors. According to institutional practice, age (>50 yr), positive LVSI, tumor size (>2 cm) and lower uterine segment involvement were considered potential adverse risk factors. Adjuvant therapy strategy was as follows: Observation for stage 1A-grade 1 disease with no adverse risk factors; brachytherapy alone for stage 1A-grade 1 disease with one of the risk factors, stage 1A-grade 2-3 disease with no risk factors and stage 1B-grade 1-2 disease with no risk factors; external beam pelvic radiotherapy for stage 1A-grade 2-3 disease with one of the risk factors, stage 1B-grade 1-2 disease with one of the risk factors, stage 1B-grade 3 disease and stage 2 disease; chemotherapy plus external beam radiotherapy for stage 3 disease; and chemotherapy alone for stage 4B disease. The chemotherapy regimen included six cycles of paclitaxel 175 mg/m^2^ plus carboplatin dosed at an area under the curve (AUC) of 5 to 6.

The surveillance practice was to follow-up patients who achieved a complete clinical remission after primary therapy every three months for two years, every six months for the next three years, and then annually. Recurrence was defined as any documented relapse of the tumor, either systemically or locally, after a disease-free interval of more than three months.

### Statistical Analysis

All analyses were performed using IBM SPSS Statistics 20 software (SPSS/IBM, Chicago, IL, USA). Binary variables were reported as counts and percentages; continuous variables were expressed as mean, standard deviation, median, and range. A multiple linear regression analysis was performed to demonstrate correlation among variables of interest. All variables were separately evaluated by a univariate analysis using the Mann-Whitney U test and Spearman’s rank correlation coefficients (r value). Variables with a p value <0.05 in the univariate analysis were selected and included in the multivariate analysis.

To define the diagnostic threshold values of ADC_min_ and SUV_max_, a receiver operating characteristic (ROC) curve analysis was performed by plotting every possible cutoff score’s sensitivity on the y-axis against 1-specificity on the x-axis. The Youden index was calculated to choose the optimal cutoff values (16). For the ROC curve, the point with the largest sum of specificity and sensitivity was chosen as a threshold. In presenting the results, sensitivity, specificity, positive predictive value (PPV), and negative predictive value (NPV) were all reported. The AUCs of ROC curves and their 95% confidence intervals (CI) were compared using the method of DeLong et al. (17).

## RESULTS

**Figure 1:**
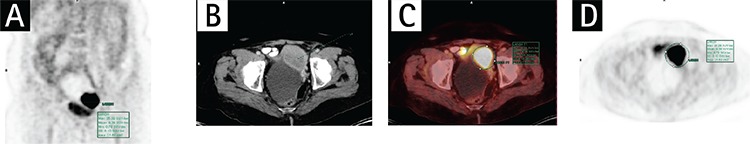
Maximum standardized uptake value of the primary tumor was measured 35,39. Endometrioid type endometrial carcinoma (grade 2, International Federation of Gynecology and Obstetrics stage 3A). A) Maximum intensity projection image. B) Transaxial computed tomography image. C) Transaxial positron emission tomography+computed tomography fusion image. D) Transaxial positron emission tomography image of the primary tumor

**Figure 2:**
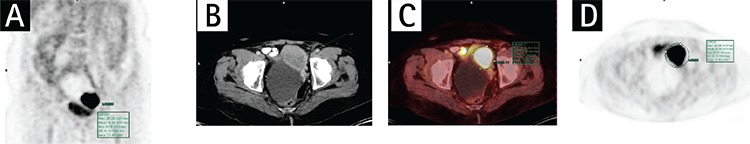
Maximum standardized uptake value of the primary tumor was measured 23,29. Non-endometrioid type (serous) endometrial carcinoma (grade 3, International Federation of Gynecology and Obstetrics stage 3C). A) Maximum intensity projection image. B) Transaxial computed tomography image. C) Transaxial positron emission tomography+computed tomography fusion image. D) Transaxial positron emission tomography image of the primary tumor

**Figure 3:**
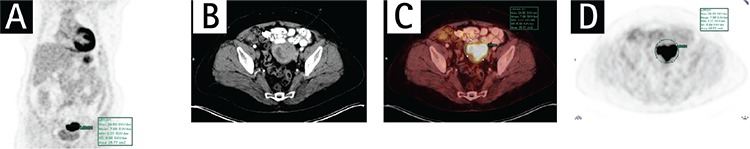
Maximum standardized uptake value of the primary tumor was measured 29,50. Endometrioid type endometrial carcinoma (grade 1, International Federation of Gynecology and Obstetrics stage 2). A) Maximum intensity projection image. B) Transaxial computed tomography image. C) Transaxial positron emission tomography+computed tomography fusion image. D) Transaxial positron emission tomography image of the primary tumor

A total of 45 patients were enrolled in the analysis. Table 1 presents the characteristics of patients. The mean age was 57.11±11.12 years. The mean ADC_min_ and SUV_max_ of the primary tumor were 0.72±0.22 and 16.54±8.73, respectively. The majority of patients (73.3%) had combined pelvic and paraaortic lymphadenectomy. The median number of pelvic LNs removed, paraaortic LNs removed, and total LNs removed (pelvic and paraaortic) were 28, 23, and 44, respectively. The distribution of the surgical stages of patients was as follows; stage 1A 21 patients (46.7%), stage 1B seven patients (15.6%), stage 2 six patients (13.3%), stage 3A three patients (6.7%), stage 3C six patients (13.3%), and stage 4B two patients (4.4%). Most of the patients (77.8%) had endometrioid histology. Deep MI (≥½) was observed in 35.5% of the patients, LVSI in 28.9%, cervical invasion in 31.1%, adnexal invasion in 13.3%, and LN metastasis in 17.8%. During the median follow-up period of 20 months (range, 7.5-30.5 months), six patients (13.3%) experienced disease recurrence with a median time to recurrence of 6 months (range, 4.5-15.5). Three examples of such cases are shown in Figure 1, 2, 3.

The results of multiple linear regression analysis were summarized in Table 2. In univariate analysis, while the potential factors associated with ADC_min_ were age (p=0.006), deep MI (p=0.021), and LVSI (p=0.015); the potential factors associated with SUV_max_ were age (p=0.022), stage (p=0.003), tumor size (p=0.001), deep MI (p=0.001), LVSI (p=0.007) and number of metastatic LNs (p=0.049). However, only the deep myometrial invasion remained to be an independent variable associated with ADC_min_ (p=0.007) as well as SUV_max_ (p=0.024) after adjustment for other confounders in multivariate analysis. There was a significant but moderate and negative correlation between the ADC_min_ and SUV_max_ (r=-0.518, p<0.001).

**Figure 4:**
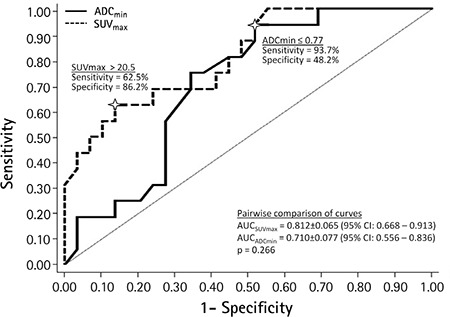
Receiver operating characteristic curve analysis for the diagnostic value of minimum apparent diffusion coefficient and maximum standardized uptake value of the primary tumor in predicting deep myometrial invasion ADC_min_: Minimum apparent diffusion coefficient, SUV_max_: Maximum standardized uptake value, CI: Confidential interval

Optimal cutoff values of ADC_min_ and SUV_max_ for predicting deep MI were found to be ≤0.77 (93.7% sensitivity, 48.2% specificity, 50.0% PPV, and 93.0% NPV) and >20.5 (62.5% sensitivity, 86.2% specificity, 71.0% PPV, and 81.0% NPV), respectively; although the comparison of two diagnostic tests revealed no statistical significance [AUC-ADC_min_=0.812 (95% CI: 0.668-0.913), AUC-SUV_max_=0.710 (95% CI: 0.556-0.836); p=0.266], (Figure 4). Moreover, the combination of two biomarkers (ADC_min_ ≤0.77 and SUV_max_ >20.5) failed to improve the diagnostic accuracy (56% sensitivity, 86.2% specificity, 69.2% PPV, and 78.1% NPV).

## DISCUSSION

In the current study, we investigated the correlation between various clinicopathologic features and SUV_max_ and ADC_min_ of the primary tumor in patients with EC. The study provides evidence that the depth of MI is the sole clinicopathologic feature independently associated with SUV_max_ as well as ADC_min_. The combination of one of these biomarkers with intraoperative frozen-section examination may offer better prediction of deep myometrial invasion, and thereby selection of patients for an extensive surgery.

Several studies have evaluated the predictors of extrauterine tumor spread in EC patients, and most studies reported age, tumor grade, myometrial invasion, LVSI, and tumor histology as potential risk factors (6). With respect to these factors, there are various suggested risk assessment models in the current literature (10,18,19,20); however, the majority of these models are based on the results of preoperative biopsy and intraoperative frozen-section examination, which have been shown to be prone to underestimation of tumor grade and MI in 15% to 20% of patients (11,21). Although a comprehensive staging surgery still remains the most reliable approach for determining extrauterine tumor spread, it is evident that there is a need to develop novel preoperative risk assessment strategies to avoid systematic overtreatment in patients with EC.

Emerging data indicates that ADC_min_ value derived from DW-MRI and SUV_max_ derived from ^18^F-FDG PET/CT may have a potential role in preoperative assessment of patients with EC (14). DW-MRI can visualize the microscopic movement of extracellular water protons, which allows discrimination of tissues according to their cellularity and fluid diffusivity (22). The diffusivity can be quantified by calculating the ADC_min_ value. SUV_max_ is a measure of glucose metabolism rate, which is also correlated with the cellularity of the tissue. When compared to benign lesions, malignant tumors show higher cellularity, and thereby lower ADC_min_ and higher SUV_max_ values (23,24). Although various studies suggested a possible relationship between the SUV_max_ and ADC_min_ of the primary tumor and tumor characteristics such as grade, myometrial invasion, stage, recurrence, and survival (13,14,25,26,27), uncertainty remains regarding the true magnitude and structure of these relationships as there are limited data that compare both parameters in the same study group.

In the single study investigating the relationships of SUV_max_ and ADC_min_ obtained from a preoperative evaluation with ^18^F-FDG PET/CT and DW-MRI to clinicopathologic characteristics in patients with EC, Nakamura et al. (14) reported the data of 131 patients, with a median time to follow-up of ~20 months. The authors found that low ADC_min_ values were associated with stage 3 to 4 disease (p<0.001), grade 3 tumor (p<0.001), deep MI (p=0.002), cervical involvement (p=0.001), LN metastasis (p=0.018), LVSI (p<0.001), and large tumor size (p<0.001). Although there was a significant and inverse correlation with ADC_min_ and SUV_max_ (r=-0.677, p<0.001), the SUV_max_ of the primary tumor was associated with disease-free and overall survival rates while ADC_min_ was not. The other study of interest relating to this issue in the literature was reported by Shih et al. (28). Although the authors used the data obtained from an integrated PET/MRI system, they similarly found a significant inverse correlation between the SUV_max_ and ADC_min_ of the primary tumor in 36 patients with EC (r=-0.53, p=0.001). In that study, both SUV_max_ and ADC_min_ were significantly associated with many prognostic factors; however, unlike the study by Nakamura et al. (14), the authors found no significant association between SUV_max_ and tumor grade, as well as between ADC_min_ and myometrial invasion, LVSI, and LN metastasis.

A significant inverse correlation between SUV_max_ and ADC_min_ was also evident in our study. Contrary to previous studies, we observed that the SUV_max_ and ADC_min_ values were only associated with the depth of MI among all clinicopathologic factors. Both imaging biomarkers were comparable in their abilities to estimate deep myometrial invasion. However, combining these two biomarkers resulted in a decrease in the specificity rate and NPV. It is possible that the discrepancy between our findings and those of other researchers may be due to the differences in statistics used and sample size. While the previous studies assessed the relationships between variables by using correlational statistics only, we applied a multiple linear regression analysis to determine the independent effect of each variable. This method provided controlling for the potential confounding variables.

As with all studies, the analyses presented in this paper are not without limitations. Single-institutional cohort studies, such as this one, are inherently susceptible to referral and selection bias affecting the generalizability of findings. The small sample size of our study might have caused a sampling error, limiting the power in detecting associations. A relatively short median follow-up time and lack of analysis of other potential confounders, such as comorbidities, anthropometric measurements, smoking, and biochemical markers could also be considered potential limitations.

## CONCLUSION

In conclusion, based on our results, SUV_max_ of the primary tumor derived from ^18^F-FDG PET/CT and ADC_min_ of the primary tumor derived from DW-MRI may have a role in predicting deep MI with similar diagnostic accuracies. However, the predictive performances of both imaging biomarkers do not seem high enough to support their routine use. Furthermore, the combined use of the two tests may lead to worsening of the predictive accuracies of each biomarker. Trials with a larger cohort of patients and longer follow-up data are needed for further validation of these biomarkers.

## Figures and Tables

**Table 1:**
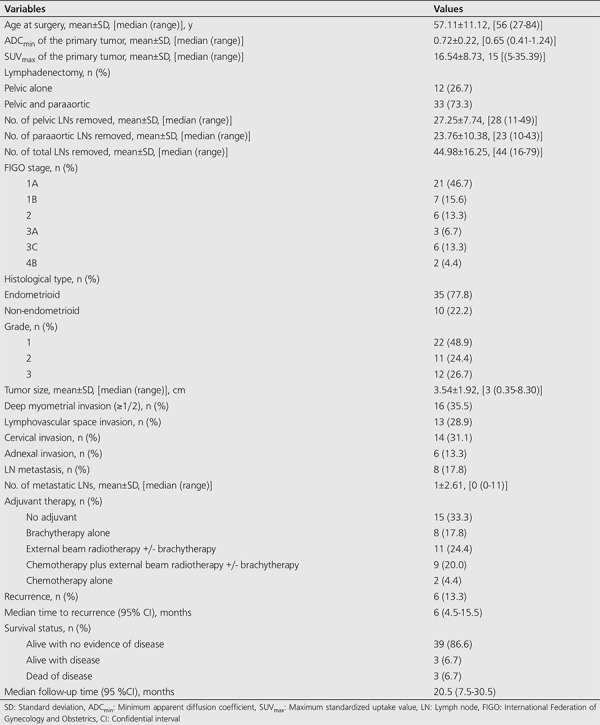
Characteristics of patients

**Table 2:**
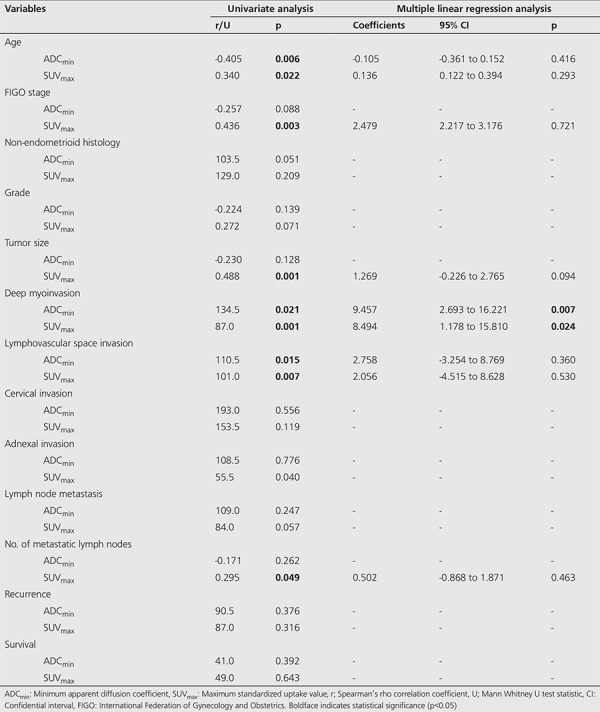
Univariate and multivariate linear regression analysis of factors associated with minimum apparent diffusion coefficient and maximum standardized uptake value of the primary tumor
